# Haemodynamic performance of neuromuscular electrical stimulation (NMES) during recovery from total hip arthroplasty

**DOI:** 10.1186/1749-799X-8-3

**Published:** 2013-03-05

**Authors:** Barry J Broderick, Oisin Breathnach, Finbarr Condon, Eric Masterson, Gearóid ÓLaighin

**Affiliations:** 1 Electrical & Electronic Engineering, School of Engineering & Informatics, NUI Galway, University Road, Galway, Ireland; 2 Bioelectronics Research Cluster, National Centre for Biomedical Engineering Science, NUI Galway, University Road, Galway, Ireland; 3 Department of Orthopaedics, Mid-Western Orthopaedic Hospital, Croom, Co, Limerick, Ireland

**Keywords:** Neuromuscular electrical stimulation, NMES, Deep vein thrombosis, DVT, Total hip arthroplasty, Lower limb hemodynamics

## Abstract

**Background:**

Patients post total hip arthroplasty (THA) remain at high risk of developing Deep Vein Thrombosis (DVT) during the recovery period following surgery despite the availability of effective pharmacological and mechanical prophylactic methods. The use of calf muscle neuromuscular electrical stimulation (NMES) during the hospitalised recovery period on this patient group may be effective at preventing DVT. However, the haemodynamic effectiveness and comfort characteristics of NMES in post-THA patients immediately following surgery have yet to be established.

**Methods:**

The popliteal veins of 11 patients, who had undergone unilateral total hip replacement surgery on the day previous to the study, were measured using Doppler ultrasound during a 4 hour neuromuscular electrical stimulation (NMES) session of the calf muscles. The effect of calf muscle NMES on peak venous velocity, mean venous velocity and volume flow were compared to resting values. Comfort was assessed using a 100mm non-hatched visual analogue scale taken before application of NMES, once NMES was initiated and before NMES was withdrawn.

**Results:**

In the operated limb NMES produced increases in peak venous velocity of 99% compared to resting. Mean velocity increased by 178% compared to resting and volume flow increased by 159% compared to resting. In the un-operated limb, peak venous velocity increased by 288%, mean velocity increased by 354% and volume flow increased by 614% compared to basal flow (p<0.05 in all cases). There were no significant differences observed between the VAS scores taken before the application of NMES, once NMES was initiated and before NMES was withdrawn (p=.211).

**Conclusions:**

NMES produces a beneficial hemodynamic response in patients in the early post-operative period following orthopaedic surgery. This patient group found extended periods of calf-muscle NMES tolerable.

**Trial registration:**

ClinicalTrials.gov NCT01785251

## Introduction

Venous thromboembolism (VTE) is the compound term used to describe the formation of deep vein thrombosis (DVT) followed by its frequent sequela, pulmonary embolism (PE). A DVT is an abnormal blood clot that develops in a deep vein. A dislodged clot can result in damage to the venous valvular system or migrate to the lungs causing a pulmonary embolism. DVT remains a serious concern among hospitalised patients with death occurring in approximately 6% of DVT cases [1]. In addition to increasing mortality, DVT also prolongs hospitalisation which increases healthcare costs [2]. The valvular destruction associated with a dislodged thrombus leads to post-thrombotic chronic venous insufficiency in 30% of cases as well as venous ulceration and the risk of recurrent DVT [3,4]. The incidence rates of DVT remain unacceptably high with rates as high as 30% in general surgical patients and 85% in orthopaedic patients without prophylaxis [5-8].

By examining the mechanisms that give rise to DVT, it also becomes apparent how it can be prevented. Virchow described the pathogenesis of DVT as a triad of factors: (i) alterations in the blood constitution or hypercoagulability, (ii) alterations in blood flow or venous stasis and (iii) vascular endothelial damage [9,10]. Two of Virchow’s criteria, venous stasis and hypercoagulability, are closely linked. Venous stasis is the slowing down or stopping of blood flow in the deep veins, typically as a result of long periods of immobility [3]. Hypercoagulability is an increase in the propensity of a clot to develop due to the shifting in the balance of circulating procoagulants and anticoagulants in favour of the former [11]. When venous stasis is present, hypercoagulability follows.

Venous stasis can be prevented by improving venous circulation. The most common method of accomplishing this is through the use of mechanical devices such as graduated compression stockings (GCS) that boost venous circulation by apply graduated compression to the lower limb. Intermittent pneumatic compression (IPC) devices are another frequently used blood flow enhancement method. IPC operates by expanding and contracting an air-tight cuff that is wrapped around the legs or feet. Mechanical devices are often administered in conjunction with anticoagulants for higher risk patients, or in place of anticoagulants in situations where anticoagulants are contraindicated [8]. Despite GCS and IPC being the most common mechanical prevention devices for DVT prevention, there are disadvantages associated with their use. Both can be hot, constricting and itchy, resulting in poor patient compliance to the prescribed treatment [12-14].

Neuromuscular Electrical Stimulation (NMES), another DVT preventative method, is the application of an electrical stimulus to motor points in the body using electrodes placed on the surface of the skin to elicit a muscular contraction. Calf muscle activation compresses the intramuscular and surrounding veins which raises venous blood pressure and forces blood back toward the heart. Furthermore, contracting the calf muscles increases lower limb blood flow in order to meet the metabolic demands of exercising skeletal muscles [15]. The increase in lower limb blood flow resulting from activation of the calf muscle pump prevents the pooling and stagnation of blood in the lower limb. NMES could be used in situations of low levels of patient mobility to artificially activate the calf muscle pump, thereby preventing the stagnation of blood and hence DVT.

Patients post total hip arthroplasty are at high risk of developing Deep Vein Thrombosis (DVT) during the recovery period following surgery [16]. The restricted hip range of motion and the necessity for bed rest contribute to lack of skeletal muscle pump activity, resulting in reduced venous return of blood from the lower extremities [13]. The use of NMES during the hospitalised recovery period on this patient group may be effective at preventing DVT by artificially activating the calf muscle pump. Previous studies have demonstrated that NMES is effective at increasing popliteal vein blood flow in healthy participants subjected to bed rest, in patients with chronic venous insufficiency and in patients who have previously undergone both hip and knee replacement surgery [17-19]. Furthermore, it has been shown that NMES does not cause hypersensitivity in patients with metal implants who are at least 3 weeks and up to 6 months post-surgery [18]. However, the haemodynamic effectiveness and comfort characteristics of NMES in post-THA patients immediately following surgery has yet to be established.

### Objectives

The hypothesis of this study is that the application of NMES to the calf muscles in the immediate hospitalised recovery period following total hip arthroplasty (THA) will significantly increase venous return. The main objectives are:

1. To establish if patients in the early post-operative period have a similar tolerance for NMES as the participants in previous studies had.

2. To determine if applying NMES to patients immediately post-THA increases venous outflow from the lower limb over resting conditions.

### Patients and methods

#### Patients

Patients who had undergone THA at the Mid Western Regional Orthopaedic Hospital, Croom, Co. Limerick, Ireland on the previous day were recruited for this study. Each patient gave their written informed consent to take part and the study was approved by the Ethics Research Committee at the Mid Western Regional Hospital, Limerick. Patients with a history of Diabetes Mellitus and Peripheral Vascular Disease were excluded from participation. Thrombo-embolus deterent (TED) stockings and the oral anticoagulant rivaroxaban (Xarelto®) were provided to each patient post-operatively.

#### Neuromuscular electrical stimulation

**Figure 1:**
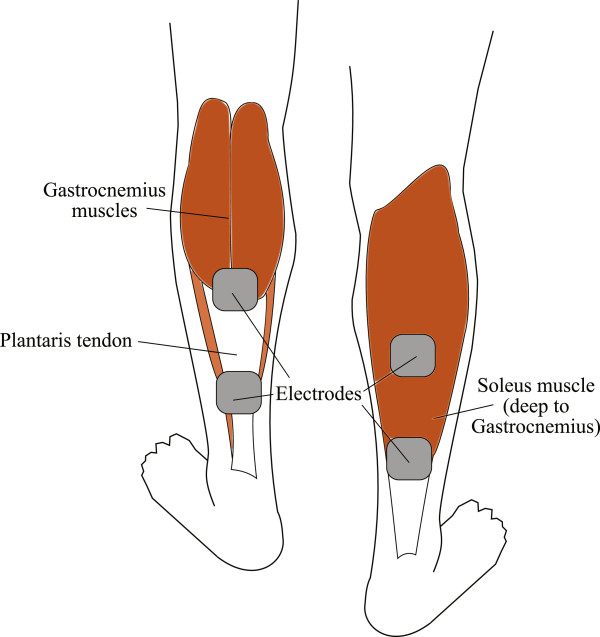
An illustration of the posterior aspect of the legs showing the position of the two NMES electrodes placed over the soleus muscles of both legs.

Two surface electrodes measuring 5 cm × 5 cm (PALS UltraStim, Axelgaard Manufacturing Co., Ltd., CA, USA) were placed over the motor points of the soleus muscles of both legs of consenting patients as shown in Figure 1. NMES was applied using a custom-built, two-channel stimulator (Duo-STIM, Bioelectronics Research Cluster, NUI Galway [20]). The stimulator was programmed to apply a biphasic square-wave with a 350 μs pulse width, an inter-pulse interval of 100 μs and a frequency of 36 Hz. A comfortable calf muscle contraction was produced using a contraction time of 1s, a ramp up time of 500 ms and ramp down time of 500 ms which resulted in a slight plantar flexion. A series of test pulses were applied initially at a very low intensity to ensure correct electrode placement and to establish that the patient was comfortable with the sensation of electrical stimulation. The stimulus intensity was then gradually increased until a noticeable contraction was observed for both legs, as indicated by a visible tightening of the soleus muscle or a slight plantar flexion. Once the patient was comfortable with the chosen stimulation amplitude, the Duo-STIM was set to run for 4 hours. Stimulation parameters were set so that stimulation was applied alternatively to each leg every 30 seconds. Both legs were stimulated despite the patient having only a single operated limb so as to compare the haemodynamic performance in the operated limb versus the un-operated limb. Moreover, both legs are susceptible to DVT during unilateral THA [21].

#### Doppler ultrasound measurements

Lower limb haemodynamic performance and patient comfort were the primary outcome measures of the study. Duplex Doppler ultrasound was used to monitor the patients’ lower limb haemodynamics using a 4–8 MHz linear transducer (LOGIQ e; GE Medical Systems). Haemodynamics were measured in both legs in the popliteal vein by placing the probe at the popliteal fossa. The authors do acknowledge that symptoms of DVT are more likely to be observed when the thrombus extends proximally into the veins of the thigh, from where it is more likely to embolise and result in PE [22]. Measurement of the femoral vein blood flow however was felt to be inappropriate in this study as non-clinical staff were involved in ultrasound measurement.

**Figure 2:**
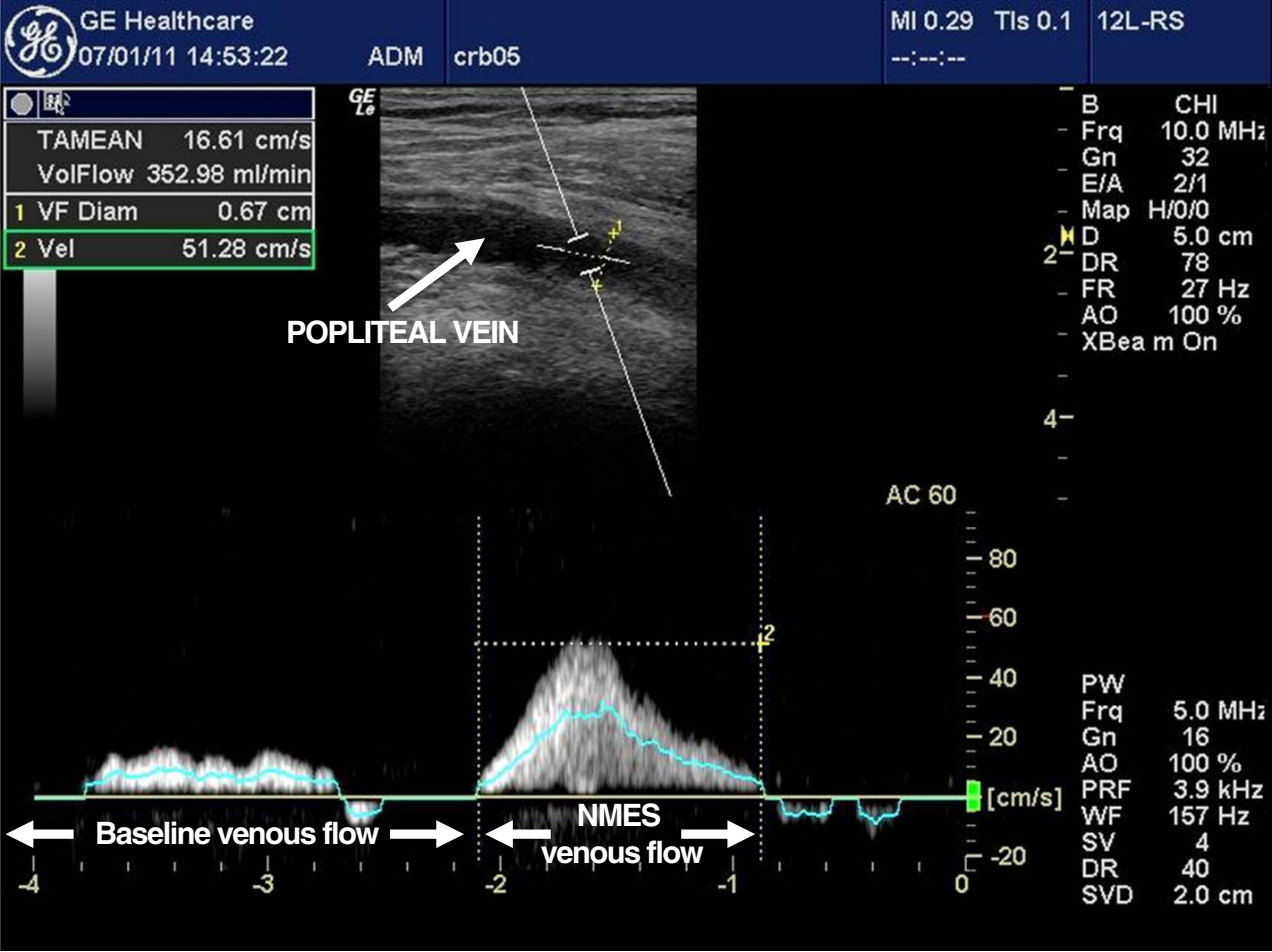
A screenshot of the Doppler machine showing baseline blood flow followed by NMES elicited blood flow.

The Doppler sample gate size was matched to the diameter of the popliteal vein. Measurements of peak venous velocity (cm/s) and popliteal vein diameter were recorded. The Doppler machine’s own software was used to calculate time averaged mean velocity (TAMEAN, cm/s) and volume flow (ml/min) (Figure 2). Each measurement was taken three times for rigour and the mean of the three measurements was used for analysis. At least one minute of rest was allowed between successive Doppler measurements. Doppler measurements were taken initially at rest, before the application of NMES. Doppler measurements were taken once again when the NMES session had commenced.

At each of 3 time points (before application of NMES, after application of NMES had begun and at the end of the protocol), comfort was assessed by asking patients to mark their level of comfort using a 100 mm, non-hatched visual-analogue-scale (VAS). A VAS of 30 mm or less was categorised as mild pain, between 31 and 69 mm as moderate pain and scores of 70 mm or greater as severe pain. The minimum clinically significant difference (MCSD) in VAS was set as an increase in scores between test stages of 12 mm [23].

#### Statistical analysis

Differences between stimulated and resting peak venous velocity, mean velocity and volume flow measurements for both the operated and un-operated limbs were analysed using the Wilcoxon signed-rank test. Differences in resting blood flow between the operated and un-operated limb were compared using the Mann–Whitney test. Differences in the increase in blood flow produced by NMES between the operated and un-operated limb were also compared using the Mann–Whitney test. VAS scores were analysed by a repeated-measures analysis of variance (ANOVA). A p*-*value of <0.05 was considered statistically significant in all cases.

## Results

Eleven patients took part in this study (4 male, 7 female) that had a mean age of 69.5 ± 8.1 years. Blood flow measurements from both the operated limb and the un-operated limb were recorded in 7 patients. Blood flow measurements from just the operated limb were recorded in 4 patients. There was no significant difference between the NMES voltage applied to the operated limb and the un-operated limb (39.7±12.3 V versus 40.5±12.5 V, p=0.68).

### Peak venous velocity

**Figure 3:**
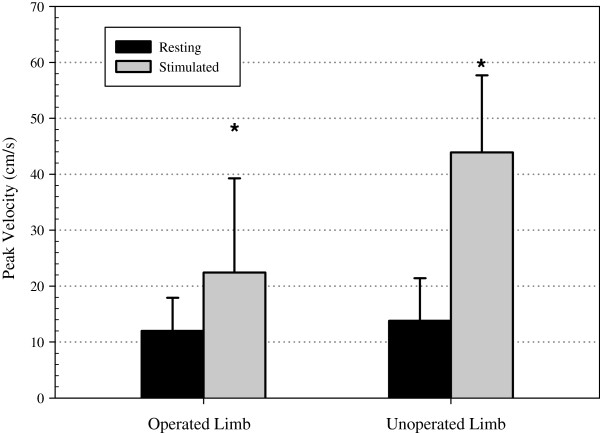
Peak venous blood flow velocity measurements due to the NMES elicited calf muscle contraction versus resting in the operated limb and un-operated limb (* P < 0.05 compared to resting peak velocities).

Figure 3 shows the resting and stimulated peak venous velocities from both the operated limb and un-operated limb. NMES produced peak venous velocities that were ~99% higher than resting in the operated limb (12±5.9 versus 22.5±16.8 cm/s, p=0.006) and ~288% higher than resting in the un-operated limb (13.8±7.6 versus 43.9±13.7 cm/s, p=0.018). Furthermore, the percentage increase in peak velocity produced by NMES in the un-operated limb was significantly larger than that of the operated limb (p=0.02). There were no differences between the resting velocities of the operated and un-operated limbs (p=0.892).

### Mean venous velocity

**Figure 4:**
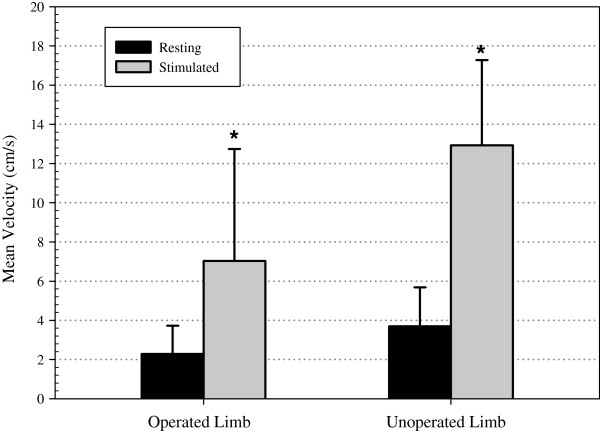
Time average mean velocity measurements due to the NMES elicited calf muscle contraction versus resting in the operated limb and un-operated limb (* P < 0.05 compared to resting mean velocities).

The resting and stimulated mean velocities from both the operated and un-operated limbs are shown in Figure 4. NMES elicited calf muscle contractions resulted in mean velocities that were ~178% higher than resting in the operated limb (2.3±1.4 versus 7±5.7 cm/s, p=0.003) and ~354% higher than resting in the un-operated limb (3.7±2 versus 12.9±4.3 cm/s, p=0.018). Moreover, there were no differences in the percentage increase in mean velocities produced compared to resting between the operated and un-operated limbs. There were no differences between the resting mean velocities of the operated and un-operated limbs (p=0.113).

### Volume flow

**Figure 5:**
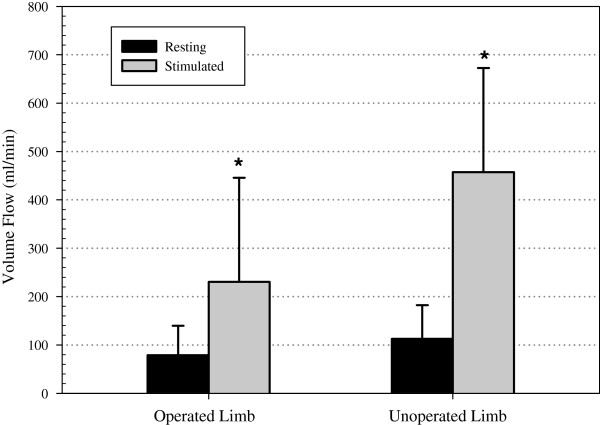
Volume flow measurements due to the elicited calf muscle contraction versus resting in the operated limb and un-operated limb (* P < 0.05 compared to resting volume flow).

There were no differences between the resting volume flow of the operated and the un-operated limbs (p=0.342). Calf NMES resulted in a 159% increase in venous volume flow in the operated limb (78.7±61.1 versus 230.4±215.2 ml/min, p=0.003) and an ~614% increase in un-operated limb (112.6±69.5 versus 457.6±215 ml/min, p=0.018) (Figure 5). There were no differences in the percentage increase in volume flow between the operated and un-operated limb.

### Assessment of comfort

**Figure 6:**
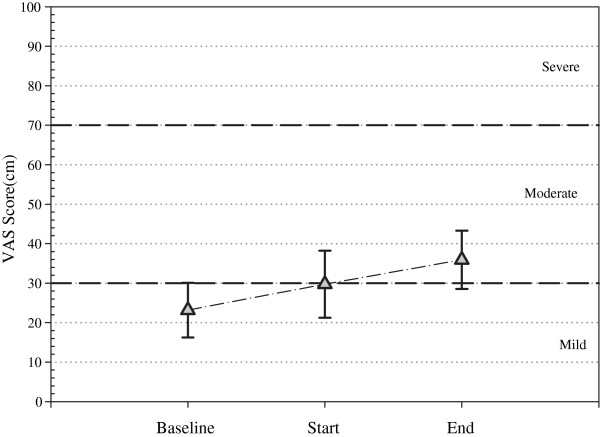
Mean VAS scores for all patients (n=11) at baseline, when NMES was started and just before NMES was finished (error bars indicate SEM).

Each patient’s individual VAS scores are summarized in Table 1 and Figure 6. The repeated measures ANOVA revealed no differences between the baseline, start and end VAS scores (p=0.211). Baseline VAS categorical scores, before the application of NMES, indicated mild pain in 7 patients, moderate pain in 3 patients and severe pain in 1. When NMES was first applied, categorical scores remained unchanged in all but one patient, whose categorical score increased from mild to moderate. At the end of the NMES session, VAS categorical scores remained unchanged in 5 patients, with 3 patients indicating an increase from mild to moderate pain and one patient indicating an increase to severe pain. All of these patients indicated an increased VAS score was greater than the MCSD. Moreover, two patients indicated a decrease in categorical score, one which was greater than the MCSD. This patient indicated a decrease from moderate to mild.

**Table 1:** Vas scores, pain category and the difference between scores before NMES (baseline), after NMES was started (start) and just before NMES was finished (end)

**Patient**	**VAS baseline**	**Pain category**	**VAS start**	**Pain category**	**Difference start-baseline**	**VAS end**	**Pain category**	**Difference start-end**
1	34	Moderate	36	Moderate	2	54	Moderate	18
2	8	Mild	10	Mild	2	23	Mild	13
3	47	Moderate	46	Moderate	−1	40	Moderate	−6
4	5	Mild	14	Mild	9	41	Moderate	27
5	46	Moderate	69	Moderate	23	7	Mild	−62
6	23	Mild	60	Moderate	37	80	Severe	20
7	3	Mild	5	Mild	2	30	Moderate	25
8	4	Mild	4	Mild	0	4	Mild	0
9	12	Mild	7	Mild	−5	10	Mild	3
10	3	Mild	2	Mild	−1	40	Moderate	38
11	70	Severe	74	Severe	4	66	Moderate	−8

## Discussion

This study showed that applying NMES to the calf muscles of patients in the early post-operative period following THA produces popliteal blood flow velocities that far exceed resting in the operated and in the un-operated limb. NMES induced a peak velocity in the popliteal vein that reached nearly four times as high as resting. NMES induced a mean velocity that was also approximately four times as high as resting and volume flow was seven times that of resting.

In as early as 1964, Doran et al. [24] demonstrated an improvement in venous return when electrical muscle stimulation was applied to the calf muscles of patients compared to when no stimulation was used. Since Doran’s study, dramatic improvements in lower limb venous blood flow have been demonstrated in healthy patients with calf NMES resulting in peak velocities of 43-120 cm/s in the popliteal vein [17,25-28]. More recent contributions to the area of NMES for blood flow assist included the investigation of the ideal pulse repetition rate on ejected blood volume from the lower limb [25] and the benefits of targeting the peroneal nerve with surface stimulation rather than targeting the motor points of the muscle directly [26].

In our study, we observed a popliteal peak velocity of ~43 cm/s in the un-operated limb which was in line with these previous studies despite implementing the study on a post-THA patient group. However, peak velocity measurements in the operated limb were approximately half that of the un-operated limb (~22 cm/s) despite there being no differences in the applied NMES intensity between limbs. It was noted that there was increased swelling in the operated leg in each instance. Manipulation of the limb intraoperatively contributes to venous stasis. In addition, postoperative immobilization after surgery is another reason contributing to the reduced venous return and thus increase the risk of DVT [22].

On the first post-operative day, electrode placement is very difficult as patients have a significantly reduced range of motion on the operated leg. NMES electrodes are generally placed with the patient in a standing or prone position which allows for the easy identification of the required muscle landmarks. While patients who are one day post-THA can be brought into a standing position, we found that it was a significant ordeal for the patient to do so for the duration required for the placement of the electrodes. Therefore, placing the NMES electrodes at this point was impractical and would inflict undue discomfort on the patient. Furthermore, the patients cannot adopt a prone position, nor can they perform a plantar flexion in order to help identify muscle landmarks. Therefore, placing the electrodes could only be done with the patient lying in a semi-supine position. The procedure required two people, one to carefully hold the leg in a slightly elevated position and the other person to place the electrodes. As an incorrect electrode placement can lead to an unsatisfactory treatment outcome [29] and can lead to an unpleasant sensory perception from the stimulus, we recommend that sufficient time and care be taken when placing the electrodes and ensure that a smooth calf muscle contraction is observed before starting the NMES treatment protocol.

VAS scores in this study showed that NMES did not significantly increase the perceived discomfort of the patient. Overall, the observed VAS scores were in line with previous observations on healthy participants and on patients with metallic hip implants [17,18]. The patient that indicated severe pain at the end of the study reported that the stimulation intensity considered comfortable at the start of the study eventually caused calf muscle fatigue toward the end of the study. This suggests that a four hour NMES session at a constant NMES intensity may be too fatiguing for patients and thus, patients should be encouraged to select a more appropriate intensity at the beginning of the NMES session. The patient who indicated severe pain before the application of NMES reported that the surgical site was the source of pain, yet the patient was happy to take part in the study. NMES did not significantly influence this patients VAS score. The same patient reported severe stiffness of the ankle on the operated limb before starting the NMES protocol which was significantly improved by the end of the protocol. A solution to this problem could be to place the electrodes on the calf muscles prior to surgery.

There are a limited number of studies examining the effect that other mechanical DVT preventative measures have on patient comfort. Tamir et al. [30] used a foot compression device on total knee arthroplasty patients and showed an average VAS score of 3.3. Another study by Anand and Asumu [31] showed an average VAS score of 6.7 when a pneumatic foot pump was applied to hip and knee surgery patients. Although these results are interesting when compared to the average VAS score in this study (3.5), no real comparison can be made due to the difference in patient groups and differences in the post-surgery care, such as analgesic type and dosage. However, if NMES is proven to provide the same prophylaxis as other mechanical DVT prevention methods, then the decision on which type of device to use may come down to patient comfort.

### Practical limitations

In addition to the difficulty in placing the electrodes, the electrode-to-lead connector was very short and had to be placed underneath the antiembolism stocking. The pressure exerted by the stocking on the connector resulted in the connector being pressed against the already swollen leg. Patients complained that this was uncomfortable and a red mark was visible on the skin once the electrodes were removed. This situation did not arise in our previous studies as NMES was never applied underneath antiembolism stockings. To prevent this occurring in future NMES studies on post-THA patients, electrodes with longer lead connectors should be used so that they are not placed under the stocking.

## Conclusion

The results of this study show a beneficial haemodynamic response to NMES in patients in the early post-operative period following orthopaedic surgery which has implications for DVT prevention and reduction of edema. These patients find the application of calf muscle NMES tolerable. We conclude that the use of NMES on postoperative orthopaedic patients can be considered as a DVT prevention method; however, the protocol adopted needs further refinement for the immediate post-operative period. Furthermore, the associated reduction in DVT with NMES use has yet to be established and thus, this paper motivates a large scale study.

## Competing interests

The authors are currently applying for a patent relating to the content of this paper.

## Authors’ contributions

BB designed the study, participated in acquisition of data, carried out data analysis and drafted the manuscript. OB provided medical input on the study design, carried out patient testing and assisted the drafting of the manuscript. FC and EM provided critical review for medical content, provided and supervised patient access and assisted recruitment of patients. GOL participated in the study design and coordination, helped to draft the manuscript and provided critical review for scientific content. All authors read and approved the final manuscript.
